# Radiologists in the loop: the roles of radiologists in the development of AI applications

**DOI:** 10.1007/s00330-021-07879-w

**Published:** 2021-04-16

**Authors:** Damian Scheek, Mohammad. H. Rezazade Mehrizi, Erik Ranschaert

**Affiliations:** 1 Vrije Universiteit Amsterdam, Amsterdam, The Netherlands; 2 School of Business and Economics, KIN Center for Digital Innovation, Vrije Universiteit Amsterdam, De Boelelaan 1105, VU Main Building A-wing, 5th floor, 1081 Amsterdam, HV The Netherlands; 3 Department of Radiology, Elisabeth-Tweesteden Hospital (ETZ), Tilburg, The Netherlands; 4 Ghent University, Ghent, Belgium

**Keywords:** Radiologists, Artificial intelligence, AI, Development, Roles

## Abstract

**Objectives:**

To examine the various roles of radiologists in different steps of developing artificial intelligence (AI) applications.

**Materials and methods:**

Through the case study of eight companies active in developing AI applications for radiology, in different regions (Europe, Asia, and North America), we conducted 17 semi-structured interviews and collected data from documents. Based on systematic thematic analysis, we identified various roles of radiologists. We describe how each role happens across the companies and what factors impact how and when these roles emerge.

**Results:**

We identified 9 roles that radiologists play in different steps of developing AI applications: (1) problem finder (in 4 companies); (2) problem shaper (in 3 companies); (3) problem dominator (in 1 company); (4) data researcher (in 2 companies); (5) data labeler (in 3 companies); (6) data quality controller (in 2 companies); (7) algorithm shaper (in 3 companies); (8) algorithm tester (in 6 companies); and (9) AI researcher (in 1 company).

**Conclusions:**

Radiologists can play a wide range of roles in the development of AI applications. How actively they are engaged and the way they are interacting with the development teams significantly vary across the cases. Radiologists need to become proactive in engaging in the development process and embrace new roles.

**Key Points:**

*• Radiologists can play a wide range of roles during the development of AI applications.*

*• Both radiologists and developers need to be open to new roles and ways of interacting during the development process.*

*• The availability of resources, time, expertise, and trust are key factors that impact how actively radiologists play roles in the development process.*

**Supplementary Information:**

The online version contains supplementary material available at 10.1007/s00330-021-07879-w.

## Introduction

Today, applications of artificial intelligence (AI) in radiology have become too important to ignore. The AI applications developed in the radiology domain are growing rapidly [1]. Besides the challenges [2], these applications increasingly enter the clinical practice and can significantly impact the radiology work [3]. In this situation, the radiologists’ role does not limit to being only the “users” of the applications. Radiologists, as domain experts who have deep insights into medical image diagnosis, need to actively participate in the development process [4].

The literature on AI development in general [5], and in the domain of radiology in particular, has proposed various roles that radiologists can and should play during the development of AI applications. As domain experts, they have the tacit knowledge, which can lead the AI developers towards medically relevant use cases [2, 6]. They can advise developers on how their tools can effectively contribute to the radiology work. At the same time, by being present in the development process, radiologists can better understand the principles of AI systems and identify the potential pitfalls and limitations of these applications [4]. Furthermore, including radiologists in the development can enhance the trust in AI applications, as well as the acceptance of these systems among radiologists [7]. A few studies suggest that we need to dig deeper into the development steps to better understand how radiologists can contribute to each step (see Table 1).

**Table 1:** How radiologists can contribute to AI development process

Step	Definition	How radiologists can contribute
1. Defining use case and the conceptual design	Defining clear, clinically relevant use case to achieving specific outcomes (e.g., increasing the speed, accuracy, and efficiency) and translating it into the conceptual design of the solution	Guiding the developers towards clinically relevant use cases (what problem to focus on) and how their solutions can potentially be used by radiologists [6]
2. Data sourcing and curation	Collecting, selecting, cleaning, and organizing the data that is needed for the training and validation of the algorithm	Sharing their data (images and scans), thus being the connection between the available data in the medical world and the AI vendor [8]
3. Labeling and establishing the ground truth	Defining the ground truth and (in case of supervised learning) labeling data	Radiologists as domain experts act on labeling the medical data and are consulted for establishing the ground truth [8, 9]
4. Training the algorithm	Configuring the algorithm (e.g., setting the parameters) and training it	[No specific role of radiologists is currently suggested in the literature]
5. Testing and validating the AI application	Using appropriate and dedicated (reference) datasets to validate trained algorithms and ensure their accuracy and generalizability to clinical cases	Checking the results of the algorithm to be accurate and stable and radiologists can trust them [10, 11]

Despite these suggestions, we still have a limited understanding of how these roles *actually* happen in the real world and whether radiologists play other roles in the development of AI applications. We also need to investigate how the roles of radiologists may change depending on the organizational conditions (e.g., between small startups versus large companies).

In this article, we examine the various roles of radiologists during the development of AI solutions, across eight companies, specializing in the development of AI applications for the field of radiology. We show that radiologists play a wider range of roles than what the current literature proposes. These roles, which occur in different steps of the AI development, are contingent to organizational conditions, such as the size of the organization, the background of the company, and the forms of interactions with radiologists.

In the next section, we describe how we conducted our study. Then, we report the roles of radiologists in various steps of AI development. Finally, we discuss the implications of our findings for the radiology community and their participation in the development of AI.

## Methods

We followed a multiple case-study approach [8] to capture the *diversity* of the AI developers and the ways they interact with radiologists in the development process. We followed the principle of “maximum variety” [8] (see Table 2). The inclusion criteria for selecting cases were based on maximizing the variety of the cases in terms of (1) company size (large, multinationals to small startups); (2) technological and business backgrounds (e.g., dedicated to AI development and general corporations producing medical products); and (3) geographical locations (representing distinct economic, legal, and cultural contexts).

**Table 2:** Overview of the selected AI vendors (ordered based on company size)

Company	Foundation year	Location	Company size (no. of full-time employees)	Company background	AI application (under development)	Radiologists involvement
Large multinational with diverse medical products	1847	New Jersey, USA	50.000	Large company that was originally founded from a medical background and currently tries to enhance their already existing physical medical products by implementing AI	AI application detects lung nodules on CT scans *Algorithm: Deep Learning*	*Employment:* both full-time and part-time contracts *Status:* workers, researchers *No. of radiologists involved:* ~1000 radiologists
Large multinational from China	2014	Beijing, China	300	Company was created from a medical background from which they aim to enable doctors with higher efficiency and better diagnosis for patients through medical artificial intelligence	The application detects pneumonia cases in an accurate and timely manner, lesion quantifications, detects tiny lung nodules, tuberculosis from chest radiograph, multiple functioning for X-ray image detection, accurately detects bleeding area from CT stroke images and CT bone scans *Algorithm: Deep Learning*	*Employment:* part-time contracts and freelance assignments *Status:* researchers, consultants *No. of radiologists involved:* around 15 (an entire department is allocated to the radiologists)
Medium-sized established AI vendor from Canada	2007	Calgary, Canada	144	A medical perspective has been the drive and background of this company, which enables radiologists to complete effective and precise analysis through artificial intelligence	The application assesses coronary artery disease using cardiac CT and detects cardiac abnormalities through MRI *Algorithm: Deep Learning*	*Employment:* freelance assignments *Status:* medical consultants *No. of radiologists involved:* radiologists only work for the company on a project basis. Projects could involve around 10–15 radiologists
Medium-sized startup from California, USA	2013	CA, USA	60	Company founded from a technological perspective. Company tries to provide every care provider with the skills from highly trained radiologists through technology	A point-of-care ultrasound system that helps healthcare providers, even those with no experience, to conduct quick and accurate ultrasound exams at point of care of the cardiac functioning *Algorithm: Deep Learning*	*Employment:* freelance assignments with the company and external advisors *Status:* medical consultants, researchers *No. of radiologists involved:* two medical consultants within their team; several radiologists depending on the size of the project
Small established AI vendor from Israel	2006	Haifa, Israel	45	Company builds vendor-independent images enhancements for diagnostic imaging. Company is built from a technological perspective	The application “enables the use of fast MRI protocols on MRI scanners of any vendor and model, by substantially increasing SNR and image quality” *Algorithm: Machine Learning*	*Employment:* radiologists are not part of the permanent team within the company; are only asked to give advice on certain concepts and products. *Status:* medical consultants *No. of radiologists involved:* depending on the project and kind of idea or product that are asked to be involved with
Small Dutch Startup	2012	Rotterdam, The Netherlands	35	Medically centered company, which tries to use artificial intelligence to empower the radiologists by delivering fast, objective, accurate, and insightful medical reports	Detects prostate cancers from MRI images *Algorithm: Deep Learning*	*Employment:* both part-time and full-time contracts *Status:* medical consultants, researchers *No. of radiologists involved:* two full-time and around 12 part-time employees
Small, established AI vendor in the Netherlands	2014	Nijmegen, The Netherlands	32	Company with a medical perspective from which it tries to use artificial intelligence to detect cancers earlier and thus improve breast cancer survival rates	Breast cancer detection and diagnosis for 2d and 3d mammography *Algorithm: Deep Learning*	*Employment:* part-time contract *Status:* medical consultants, researchers *No. of radiologists involved:* around 7 part-time employees, depending on the ongoing projects
Small Startup in Lithuania	2017	Vilnius, Lithuania	11	Company started from a technological background from which it tries to implement this to create easy-to-use medical products for everyday clinical practice	A fully automatic computer-aided diagnosis (CAD) chest X-ray solution. It identifies chest X-ray images with no abnormality and produces preliminary reports *Algorithm: Deep Learning*	*Employment:* part-time contracts. Radiologists as one co-founder *Status: m*edical consultants, researchers, and co-founder *No. of radiologists involved:* around 7 part-time employees, depending on the ongoing projects. Co-founder as part of the permanent team.

We collected data through semi-structured interviews with key managers and technical employees, who were involved in the development projects (see Appendix 1 for the interview protocol). Qualitative interviews enabled us to dig into when, how, why, and under which conditions radiologists played certain roles in each case [9]. We conducted 17 interviews, taking 60 min on average^1^. All the interviews were voice-recorded and transcribed verbatim. We also collected rich information about each company and the development projects based on public and internal documents (see Table A1 for an overview of data). Initially, several radiologists were consulted about their opinions on this research, and current practices of development for artificial intelligence within radiology. These radiologists were either employed by or affiliated to the companies. These consultations happened in an informal setting and offered background information about the roles of radiologists.

To systematically analyze data, we followed four steps. First, we integrated the data from the interviews and the documents into a detailed case-study report for each case. Second, we coded each report based on the inductive thematic analysis approach [10] to identify various roles of radiologists in the five steps of the development. We used the list of roles discussed in the literature (Table 1) as the initial codebook, yet openly explored other roles in our open coding. Third, for each role, we identified the tasks, official functions, and the forms of interaction between radiologists and the development team. Fourth, we compared the cases based on the roles that radiologists played in different steps to examine what conditions impacted when and how each role occurs.

## Results

**Fig. 1:**
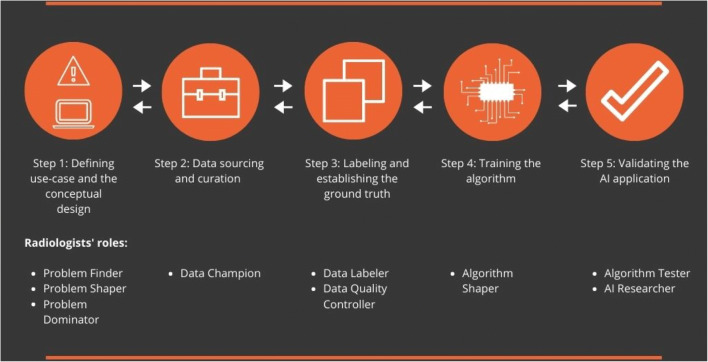
The overview of the radiologists’ roles in AI development steps

Across the companies, we identified 9 roles that radiologists played in different steps of the development process, which we describe in the following section (see Table 3 and Tables A2-A7). Figure 1 offers an overview of the radiologists' roles in different development steps

**Table 3:** Roles of radiologists during the development steps

Role	Description	How and when the role happened
Step 1. Defining use case and the conceptual design		
Problem finder	Radiologists share their knowledge and ideas to define clinical problems, needs, and use cases; but they are not in charge of making the final decisions	• By hands-on radiologists, experienced in medical practice • Often as external experts (mainly to give their expert opinion) • Often as limited, occasional consultations (limited interaction / collaboration with the development team).
Problem shaper	Radiologists are consulted to advice and give feedback on the medical problem and how the developers approach it; but they are not involved in making final decisions	• Predominantly present in companies with a medical background • Through long-term relations with radiologists (often as part-time affiliates and sometimes in-house radiologists) • Involves systematic, continuous interactions with the development team
Problem dominator	Radiologists collaborate in defining and shaping the problem and make decisions regarding the definition of the clinical problem and the requirements of the application	• Present in large companies with extensive resources and staff • Through formal (full-time) position in the company • Formal responsibility in the development team • Often with extensive knowledge of AI and its applications
Step 2. Data sourcing and curation		
Data champion	Radiologists identify relevant data and use their professional relations to get the data needed for the development	• Present in large companies with extensive resources and staff • Often when companies have strong medical background • In other cases, happened limitedly through casual consultations
Step 3. Labeling and establishing the ground truth		
Data labeler	Radiologists annotate and label the data for the purpose of training the algorithm	• A common role across companies • Challenge of finding and paying for experienced radiologists • Sometimes using radiologists for the entire labeling and sometimes using alternative approaches such as using semi- or unsupervised algorithms, using automated tools for labeling, and training non-radiologists to do the labeling
Data quality controller	Radiologists check the quality and process of labeling data (which is done by computational systems or trained employees)	• Present in companies with extensive resources and development projects • Often as in-house experts, involved in the development • Effective for companies with large-scale and extensive development projects
Step 4. Training the algorithm		
Algorithm shaper	Radiologists give feedback on the understandability and accuracy of the outcomes of the algorithm for adjusting the training of the algorithm (e.g., selecting training datasets or tweaking parameters)	• Often through casual consultations with external experts • Sometimes as an iterative participation (not later at the end of the training)
Step 5. Validating the AI application		
Algorithm tester	Radiologists test and validate the outcomes of the AI solution on its performance in various (complex) cases and under medical conditions.	• Present in almost all the cases • Often through in-house or affiliated radiologists who have a close collaboration with the vendors • In both clinical and non-clinical settings • Sometimes via “trial” versions offered to radiologists as potential users (to test it on their local data)
AI researcher	Radiologists conduct (scientific) research on the performance of the AI solutions to produce scientific evidence and legal documents (e.g., for approval procedure)	• Through in-house (only in one company), but often via external collaborations (conducting joint research) • Often formalized around the approval process (e.g., FDA, CE marked) • Involves the publication of scientific papers • Active in cases with a strong medical background or connection with academic/research institutes

### Step 1: Defining use case and the conceptual design

In the first step, developers define the medical use case: a problem to be solved and certain improvements to be achieved in radiology work. The use case should be medically relevant and offer a viable solution that can be implemented and worked with. We observed three roles of radiologists in this step. First, as *problem finders*, radiologists can initiate the idea of the use case by identifying a particular problem, need, challenge, or improvement, which can be supported, expedited, or fully automated by AI solutions. Second, radiologists may be engaged after the definition of the problem, to offer their insights and feedback on the potential tool’s functionalities, features, considerations, limitations, and challenges of the idea (*problem shaper*). Third, radiologists sometimes become the dominant actor in making the final decisions on requirements and features (*problem dominator*). We observed that these three roles are often based on occasional consultations, instead of continuous, ongoing interactions. Although radiologists were involved in the definition of the use case, they were often absent in specifying the conceptual design of the solution, such as defining the operations, designing the interface, and determining how to integrate the application to the radiology workflow.

### Step 2: Data sourcing and curation

Relevant, rich data, in the forms of images and scans, should be collected and prepared in order to train the algorithms and test them. It is crucial for the developers to recognize the relevant types of data and have a deep understanding of their attributes (what information is there), as well as the limitations and challenges of sourcing and working with them (e.g., the missing parameters and the biases in the data). Radiologists, who have the knowledge of medical data (especially medical images), can play an important role in finding and selecting relevant, rich data sources. In addition, the sourced data need to be checked, wrangled, and organized in such a way that can be fed into the algorithms (data curation). For that, data engineers consult radiologists to ensure that their curation of data does not violate the informational richness and accuracy of the data (*data champion*).

Most of the companies occasionally consulted external radiologists for sourcing and curating data (e.g., through their connections with medical institutes). Nevertheless, large companies had resources to hire radiologists to play this role. Radiologists were able to use their professional relations with PACS managers, and data warehouse managers in radiology departments, to get the data that are needed for the development of the AI solution. Sometimes, they had to decide how to collect and curate data. They also facilitated the interaction with privacy officers and ethical committees for ensuring secure and eligible handling of data.

### Step 3: Labeling and establishing the ground truth

Before training the algorithm, the acquired data should be labelled in order to set a performance standard for the algorithm for (semi-)supervised algorithms. Next to labeling, developers need to establish the ground truth, against which they can assess the accuracy and performance of their algorithm. AI vendors considered the roles of *data labeler* to be played by radiologists. Experienced data labelers came forward from long-lasting relations with medical institutions. Sometimes, vendors used automated techniques or used trained coders (who are not radiologists) for labeling data. These trained labelers were part of the technological team (e.g., software developers, data scientists), who were already familiar with the field of radiology and with the conceptual design of the AI application. This was mostly the case for small vendors, who had limited resources to hire radiologists for labeling. Then, they relied on radiologists as *data quality controllers* who checked the labeled data for accuracy and consistency and resolved the complex cases.

### Step 4: Training the algorithm

Once the data is curated and labelled, they are used for training the algorithm. The algorithm learns how to recognize certain patterns through detection, classification, and segmentation of the medical cases (e.g., detecting cancerous tumors). Training should be based on the datasets that consist of a wide range of representative cases (e.g., different types of patient profiles). Training is often performed by data scientists and programmers who select the type of algorithm, configure it, set the parameters, define the thresholds, execute the various iterations, and decide on stable, accurate configurations. These decisions are based on certain assumptions about how medical practice should take place and what is a relevant, valid result. There, radiologists played the role of *algorithm shaper*, by offering their insights and feedback on whether the technical decisions are aligned with the medical practice (e.g., are the thresholds consistent with the medical categories) and whether the results of the training are accurate, correct, and understandable from the perspective of domain experts (e.g., how to show the different classifications of lung nodules to be medically meaningful).

### Step 5: Validating the AI application

After training, the developers need to ensure that the algorithm is accurate and stable when it is used on broader datasets that represent the diversity and complexity of the real-world settings (validation) and can be effectively worked with under the clinical conditions (testing). During this step, developers need to critically check the algorithm and its application based on feeding multiple, heterogeneous datasets. For that, radiologists perform the role of *algorithm tester* by suggesting how to validate the algorithm on real-life datasets (e.g., different patient profiles and different modalities), checking the biases and sensitivity of the algorithm, examining the false-positive and false-negative cases and identifying common issues, testing the algorithm on complex cases, and specifying the boundary conditions and limitations of it. Since there is no universal standard database and procedure for validating algorithms, radiologists play a crucial role in defining the composition of validation datasets and the procedures that should be followed to ensure an accurate and mindful validation. All these enable the technical team to revise the application and algorithm in order to reach the expected performance. In addition, vendors relied on radiologists as *AI researchers* to design and execute scientific tests and produce scientifically sound evidence to endorse the performance of the AI application. In this role, they are also active in writing scientific publications (especially when the development is in the research phase) and crafting scientific sections of the legal documents (when the application is going to be approved, e.g., by FDA for commercial use).

## Discussion

Looking across the companies and different development steps, we see that radiologists play a diverse range of roles.

### Beyond annotating and labeling roles

We see that radiologists play several other roles than merely labeling data. In fact, companies are increasingly relying on other ways to annotate their data than only recruiting radiologists (e.g., using semi- or unsupervised algorithms, using trained employees to annotate data, using automatic tools for data labeling, crowdsourcing the labeling). Hence, companies can benefit from the scarce, valuable knowledge of radiologists as domain experts in other steps of the development. Many of these roles reappear along the way of using AI. For instance, evaluation and validation of AI tools is not a one-time event, but an ongoing process, since regular updates of the software and using new types of equipment require revalidation of the algorithm. Radiologists should define how the tools can and should be evaluated in the clinical environment.

### More than only offering expertise

Next to offering their expertise, radiologists can play important roles in establishing connections between the companies and medical institutions (e.g., for getting access to data) and creating trust among their fellow radiologists. Next to their medical knowledge, their technical engagement is also essential to guide the development process. In addition, they can provide guidance with the seamless integration of an application in the radiology workflow, as well as ongoing evaluation of the AI at work. This way, radiologists can contribute to the establishment of relations with the prospective clients, the introduction of the AI solutions to the clinical practice, and the definitions of viable business cases.

### Different modes of interactions with radiologists

We see that companies deploy various modes of collaboration with radiologists. When companies prefer active collaborations and they have enough resources and significant development projects, they tend to hire radiologists as part of their development team, with dedicated roles. Sometimes, companies rely on occasional interactions with external radiologists, especially when they have limited resources to hire radiologists. In some cases, radiologists are actively engaged by continuously interacting with the development teams, although they do not have any formal responsibility. When companies *co-develop* their products with certain clients, some radiologists from the clients are engaged in the process. Even though these radiologists may not be formally affiliated to the developing company, they can play all of these roles.

### Factors influencing the active engagement of radiologists

Looking across the cases, we see that several factors influence the active engagement of radiologists in the development process. Companies with strong medical background and connection with radiology departments engage radiologists from the beginning of the development process. Nevertheless, when companies are dominated by the technological teams and have a tech-heavy background, they tend to engage radiologists limitedly and in later steps of the development. This sometimes causes major challenges down the road^2^. As organizations become more mature in the development of their products, the roles of radiologists become more diverse throughout the development process. In addition, having resources available for hiring radiologists and systematically engaging them in the development is a key factor. Especially companies cannot rely only on occasional consultations and expect radiologists to help them besides their full-time medical work. Next to the resources, the active engagement of radiologists is sometimes hampered due to limited expertise of radiologists (e.g., knowing how deep learning algorithms work or how data characteristics can impact the outcomes of the algorithms), combined with their skepticism regarding how AI applications can help them.

## Conclusions

Our study showed that radiologists play a diverse range of roles in the development process. Next to these roles, there are still further engagement opportunities for radiologists especially in shaping the production of AI applications, designing interfaces, and configuring and aligning them into radiology workflow. As more diverse databases become available and stricter quality levels are demanded for the algorithms, radiologists can play a stronger role in sourcing data and advising on the development and validation of the algorithms. We expect that radiologists play new roles as organizations mature in the development, data sources become more diverse and available, and more diverse use cases are identified.

Over time, organizations should not limit their interactions with radiologists to only occasional consultations; rather, they should actively and systematically bring the radiologists on board, with responsibilities and authorities to become part of the development team. This close collaboration is crucial for iterative co-creation process between the developers and the clients. At the same time, AI developers need to recognize various roles that radiologists can play and find ways to more effectively and actively engage radiologists all through the development steps and facilitate their engagement through various forms of collaboration (e.g., external advisor, affiliated medical specialists, internal team members, and collaborative teams between data scientists and radiologists).

For radiologists, it is important to recognize the large, diverse, and yet open field of contribution. Radiologists should proactively engage in the development process and define their roles and contributions. Not all radiologists need to become developers. Yet, they need to learn enough about the development of AI applications to be able to bring their valuable medical experience into the development process. Radiologists should know the basic principles, limitations, risks, and pitfalls, and the techniques and technologies used for developing these applications and learn how to evaluate and monitor the algorithms [11]^3^.

### Limitations and avenues for future research

Although we examined a diverse sample of companies, future studies can expand the empirical scope and diversity of the cases by comparing how radiologists play roles across geographical contexts and different modes of production (e.g., in-house development, commercial companies, and co-creation). Future studies can dig into the skills and capabilities that radiologists (need to) learn (before and) during their engagement in the development process. We can still learn from following the various career trajectories of radiologists who pursued different ways of engaging in the development of AI (e.g., as external advisers, as in-house medical experts, or as leading actors in the development). Radiologists can play important roles in the “implementation” and “use” phases of the AI systems (e.g., as local champions and AI supervisors), which deserves further investigations. Finally, it is important to study the radiology community to see how they themselves perceive and play various roles in the development of AI applications.

## Supplementary Information

ESM 1 (DOCX 31 kb)

## Abbreviations

AI: Artificial intelligence
CAD: Computer-aided diagnosis
CT: Computed tomography
FDA: Food and Drug Administration
MRI: Magnetic resonance imaging
PACS: Picture Archiving and Communication System

## Footnotes

1 We initially contacted 100 companies active in developing AI, but given the need for collecting rich data, we managed to study 9 of them (partly impacted by the COVID-19 situation). Despite this pragmatic constraint, we kept adding companies to ensure that we do not see any new role of radiologists (qualitative saturation).

2 As stated by the R&D manager of a small established AI vendor in the Netherlands: “we have, of course, ran into problems when developing. Not that we were completely wrong, but, it could have been done better if we used the user feedback from the beginning.”

3 As a starting step, see the online training on “Fundamentals of Machine Learning for Healthcare,” from Stanford Uni, on the basics of machine learning for radiologists.

## Declaration

### Guarantor

The scientific guarantor of this publication is Dr. Erik Ranschaert (e.ranschaert@etz.nl).

### Statistics and biometry

No complex statistical methods were necessary for this paper.

### Methodology

• multicenter study (case studies are from different countries)
