# Supplementary material for: Radiologists in the loop: the roles of radiologists in the development of AI applications
Source: Eur Radiol. 2021 Apr 16;31(10):7960–8. doi: 10.1007/s00330-021-07879-w (PMC8050223; doi:10.1007/s00330-021-07879-w)
Supplement: Supplementary file 1 — (DOCX 31 kb) [file 330_2021_7879_MOESM1_ESM.docx]

**Appendix 1: Interview protocol & collected data**

**Appendix 1. Interview Protocol**

**Introduction**

- Purpose of our interview
- Specify the company/developer
- Specify the technology that they are developing/using
- Voice Record Consent
- Anonymity clarification
- Overview: I want to get your perspectives on four main topics: A, B, C & D

**Topic A. History of the company/ Company profile**

- Could you tell me more about yourself? - what is your function within the organization? How long have you been a part of this company?
- What type of products/services/software have your company developed
  - Follow up: what products/services/software are currently under development which you can share with me?
- How has the company decided to develop and implement AI in radiology?
- What has been the company’s biggest challenge thus far in the development and implementation process of your product/service/software? How did your organization manage to deal with this?
- if applicable: your company operates on a world wide basis. Do you see any difference in the values of your company and the values of a foreign country that you operate in?

**Topic B. Development and implementation process**

- How would you best describe the overall development process of the AI application within your organization?
- Who are the key actors within this development process?
  - Are there any actors that you would like to get involved who are currently not part of the development process? Are there any that you would like to get more involved? - Could you please explain to me why they are a part of the development process or why they are not.
- Which processes within the development and implementation of AI do you think are the most crucial for the successful implementation of your product/service? Why did you choose these as the most crucial?

**Topic C. Collaboration with radiologists**

- How are radiologists involved in the development process of your AI application?
  - follow up: if so, at which points of development?
  - What are the main challenges when working together with radiologists during the development process?
  - To what extent does the involvement of radiologists complicate the process of development?
  - Could you specify for which part of the development process requires or sees joint-decision making, co-development or exchanging of ideas with radiologists? and could you also mention the moments when the radiologists are asked for their expert opinion?
- How does your product/service influence the skills and competencies of radiologists? (give example/list of competencies/skills)
  - follow up question: what skills and competences should radiologists need to be able to use your product/service/technology to its full capabilities?
- There are debates around the involvement of radiologists, some say that they should be involved due to their expertise and some say that their involvement is not needed as ML and DL can take over their tasks such as labelling. How does your company deal with this?
- How does your product/service enhance the workflow of radiologists? If so in what ways?
- To what extent does your organization work with radiologists? If applicable, does your organization want to strengthen this collaboration in the future and in what way would you do this?
- What part of the development process sees the most collaboration with radiologists? And why?
- How would you describe the current relationship with radiologists?
  - follow up: for how long have you been working with these radiologist(s)?
  - follow up: has anything changed during this relationship
- When you decided to incorporate radiologists into the development and/or implementation process, how did/do they respond to such a request? Has there been a lot of reluctance or acceptance amongst radiologists?
  - Follow up: what do you think has caused such behavior?
- How would you describe the initial reaction of radiologists when they interact with your product/service? Does this change over time?
- After the implementation of your product/service, how can radiologists give feedback to your organization about your product/service in practice?
  - follow up: Are you satisfied with the amount of feedback you have received thus far?
  - Do you find this means of giving feedback effective enough?
  - Radiologists have the opportunity to give feedback, does this slow down the process of development?
  - Do you have any ideas on how this could be improved?
  - Is there anything else that would be needed to further enhance your collaboration with radiologists?
- Are there any other medical professionals involved in the development and implementation process?

**Topic D. AI and the future of radiology**

- Where do you see the development of AI in radiology go in the future?
- How do you see the company and its products/services represent itself in the future in the field of radiology?
- What are the long and short term development goals of your organization in regards to the development of AI in radiology?
- Do you think that radiologists can be replaced by AI in the future? Why do you think so or why don’t you think so?
- How do you think the skills and competencies of radiologists may have to change in the future in regard to the further implementation of AI in radiology?

**Closing**

- Are there any other things that you would like to know?
- Is there anything else that you would like to share on this topic?
- Follow-up; staying connected.
- Recommendations: articles, books, people, events
- Appreciation

Table A1. Information on the interviewees and secondary data

| **Company** | **Interviewees’ expertise** | **Secondary Data** |
| --- | --- | --- |
| Large multinational with diverse medical products | Senior AI Software Engineer, a Senior Data Scientist and a  Radiologist (informal) | Annual report, company website, LinkedIn company profile, news article(s), secondary interview(s), scientific paper(s), documentation from their medical and scientific partners |
| Large multinational from China | Implementation Engineer and a Radiologist (informal) | Annual report, company website, LinkedIn company profile, news article(s), secondary interview, scientific paper(s), TED talk |
| Medium-sized established AI vendor from Canada | Director of Research and Advanced Technologies Development | Annual report, company website, LinkedIn company profile, news article(s) |
| Medium-sized startup from California, USA | Machine Learning Engineer | Annual report, brochure, company website, LinkedIn company profile |
| Small established AI vendor from Israel | CEO | Company website, LinkedIn company profile, news article(s), secondary interview, scientific paper(s) |
| Small Dutch startup | Strategic Marketing and Communications Manager | Company website, LinkedIn company profile, news article(s), secondary interview, scientific paper(s), social media posts (LinkedIn, Facebook and Twitter), ECR seminar notes, documentation from affiliated university hospital |
| Small, established AI vendor in the Netherlands | Head of Research and Development | Company website, LinkedIn company profile, news article(s), scientific paper(s), documentation from their medical and scientific partners |
| Small startup in Lithuania | Co-Founder (radiologist) and a Radiologist (informal) | Company website, LinkedIn company profile, news article(s), secondary interview, scientific paper(s), social media posts (LinkedIn, Facebook and Twitter) |

**Appendix 2: Details on the roles of radiologists in AI development steps**

Table A2. Radiologists’ roles in ‘defining the use case and the conceptual design’

| **Role** | **Description** | **Tasks involved** | **Observed in** |
| --- | --- | --- | --- |
| Problem Finder | Radiologists share their knowledge and ideas to define clinical problems, needs, and use cases; but they are not in charge of making the final decisions. | - Radiologist(s) attend physical and online meetings with the product team - Radiologist(s) use different forms of (online) communication to directly, in either an informal or formal setting, share their newly found challenges | - Small, established AI vendor in the Netherlands - Large multinational from China - Small startup in Lithuania - Medium-sized startup from California |
| Problem Shapers | Radiologists are consulted to advice and give feedback on the medical problem and how the developers approach it; but they are not involved in making final decisions | - Radiologist(s) have to attend team meetings as employees - Radiologist(s) need to provide guidelines and feedback on the problem development through documentation or conversation | - Small established organization from Israel - Small Dutch startup - Medium-sized established organization from Canada |
| Problem Dominators | Radiologists collaborate in defining and shaping the problem and make decisions regarding the definition of the clinical problem and the requirements of the application. | - Radiologist(s) are obligated to set the expectations and requirements of the problem by participating in product development meetings - Radiologist(s) need to actively consult their network and expertise to find out the best fitting expectations and requirements, as they are part of the problem development team | - Large multinational with diverse medical product portfolio |

Table A3. Radiologists’ roles in ‘sourcing and curating data’

| **Role** | **Description** | **Tasks involved** | **Observed in** |
| --- | --- | --- | --- |
| Data Champion | Radiologists identify relevant data and use their professional relations to get the data needed for the development. | Radiologist(s) need to act as a data researcher, thus collect the data, strategize on their method(s) and work together with the development team whilst collecting the data | - Large multinational from China - Large multinational with diverse medical products portfolio |

Table A4. Radiologists’ roles in ‘labeling and establishing the ground truth’

| **Role** | **Description** | **Tasks involved** | **Observed in** |
| --- | --- | --- | --- |
| Data Labeler | Radiologists annotate and label the data for the purpose of training the algorithm. | - Radiologist(s) need to identify inconsistencies from the data (images and scans) and label them with their specific assigned disease - Radiologist(s) need to be or get familiar with certain specific data labelling software - Radiologist(s) are asked to send the labelled data back to the developer through safe and secure online channels | - Large multinational from China - Small startup in Lithuania - Medium-sized startup from California, USA |
| Data Quality Controller | Radiologists check the quality and process of labeling data (which is done by computational systems or trained employees). | - Radiologist(s) need to go through large amounts of labelled images and scans to validate them - Radiologist(s) has to annotate or correct the images and scans which have been done incorrectly | - Small, established AI vendor in the Netherlands - Large multinational with diverse medical products portfolio |

--- Figure A1 ---

Table A5. Radiologists’ roles in ‘training the algorithm’

| **Role** | **Description** | **Tasks involved** | **Observed in** |
| --- | --- | --- | --- |
| Algorithm Shapers | Radiologists give feedback on the understandability and accuracy of the outcomes of the algorithm for adjusting the training of the algorithm (e.g. selecting training datasets or tweaking parameters). | - Radiologist(s) need to go through a basic level of training to understand the basic principles of an AI solution - Radiologist(s) are then asked to solely give feedback through meetings, on the outcomes of the AI solution | - Small, established AI vendor in the Netherlands - Small Dutch Startup - Small Startup in Lithuania |

Table A6. Radiologists’ roles in ‘validating AI applications’

| **Role** | **Description** | **Tasks involved** | **Observed in** |
| --- | --- | --- | --- |
| Algorithm Testers | Radiologists test and validate the outcomes of the AI solution on its performance in various (complex) cases and under medical conditions. | - Radiologist(s) are asked to use their own unlabeled scans and images to test the performance of the AI solution - Radiologist(s) need to then deliver the performance review (documentation) to the developer - Radiologist(s) are often asked to also annotate the images and scans again on their inaccuracy or incorrectness, and send these back to the developer | - Medium-sized startup from California, USA - Medium-sized established AI vendor from Canada - Large multinational from China - Small established AI vendor from Israel - Small Dutch startup - Large multinational with diverse medical products portfolio |
| AI researchers | Radiologists conduct (scientific) research on the performance of the AI solutions to produce scientific evidence and legal documents (e.g., for approval procedure). | - Radiologist(s) are obligated to converse with the development team through meetings, to train themselves on the functioning and development of the AI solution - Radiologist(s) need to conduct a (scientific) research on this solution and throughout this process follow set guidelines - Radiologist(s) need to write down their findings and present a paper or any other agreed upon form of a document to the AI vendor | - Small, established AI vendor in the Netherlands |

Table A7. Distribution of the roles among the companies

| **Role** | Large multinational with diverse medical products | Large multinational from China | Medium-sized established AI vendor from Canada | Medium-sized startup from California, USA | Small established AI vendor from Israel | Small Dutch Startup | Small, established AI vendor in the Netherlands | Small Startup in Lithuania | **Frequency** |
| --- | --- | --- | --- | --- | --- | --- | --- | --- | --- |
| Problem Finder |  | 1 |  | 1 |  |  | 1 | 1 | 4 |
| Problem Shapers |  |  | 1 |  | 1 | 1 |  |  | 3 |
| Problem Dominator | 1 |  |  |  |  |  |  |  | 1 |
| Data Champion | 1 | 1 |  |  |  |  |  |  | 2 |
| Data Labeler |  | 1 |  | 1 |  |  |  | 1 | 3 |
| Data Quality Controller | 1 |  |  |  |  |  | 1 |  | 2 |
| Algorithm Shaper |  |  |  |  |  | 1 | 1 | 1 | 3 |
| Algorithm Tester | 1 | 1 | 1 | 1 | 1 | 1 |  |  | 6 |
| AI researcher |  |  |  |  |  |  | 1 |  | 1 |
